# Roles of Farnesyl-Diphosphate Farnesyltransferase 1 in Tumour and Tumour Microenvironments

**DOI:** 10.3390/cells9112352

**Published:** 2020-10-25

**Authors:** Nguyen Thi Ha, Chang Hoon Lee

**Affiliations:** College of Pharmacy, Dongguk University, Seoul 04620, Korea; nguyenha221294@gmail.com

**Keywords:** farnesyl-diphosphate farnesyltransferase 1, prognostic marker, lipid rafts, cholesterol synthesis, tumour progression

## Abstract

Farnesyl-diphosphate farnesyltransferase 1 (FDFT1, squalene synthase), a membrane-associated enzyme, synthesizes squalene via condensation of two molecules of farnesyl pyrophosphate. Accumulating evidence has noted that FDFT1 plays a critical role in cancer, particularly in metabolic reprogramming, cell proliferation, and invasion. Based on these advances in our knowledge, FDFT1 could be a potential target for cancer treatment. This review focuses on the contribution of FDFT1 to the hallmarks of cancer, and further, we discuss the applicability of FDFT1 as a cancer prognostic marker and target for anticancer therapy.

## 1. Introduction

Cholesterol is an essential biomolecule involved in various aspects of human health and disease [1]. Fluctuations in blood cholesterol levels have been linked to numerous diseases including hyperlipidaemia and atherosclerosis [2]. In particular, there have been many reports tying increases in cholesterol level to involvement in multiple cancers, and linking abnormalities in cholesterol metabolism to the progression of cancer [3,4]. Therefore, enzymes related to cholesterol metabolism and related biomolecules have attracted attention recently as potential targets for anticancer therapy [5]. Among them, farnesyl-diphosphate farnesyltransferase 1 (FDFT1, EC 2.5.1.21, squalene synthase) has been regarded as a likely target in cancer due to its high expression as a biomarker used for diagnosis of various cancers, although in some cancers FDFT1 exhibits lower expression [6,7]. Most studies have reported that the upregulation of FDFT1 is required for tumour progression, as cholesterol is necessary for cell proliferation, and lipid rafts for signalling transduction, invasion, and migration of cancer cells [4]. Therefore, research on inhibitors of FDFT1 is actively ongoing not only to lower cholesterol but also to use as anticancer agents. However, it is also necessary to consider the role of FDFT1 in terms of the importance of the tumour microenvironments (TME), which has been recognized due to recent immune checkpoint blockers. Besides, new ideas are needed to explain why FDFT1 appears to act as an oncogene in some cancers and as a tumour suppressor in other cancers.

In this review, we outline the role of cholesterol in cancer and, in this context, look at the role of FDFT1 in both tumours and TME. We present a new explanation of the issues related to cholesterol and the role of FDFT1 in cancer through effects mediated by, and the mechanism of action of FDFT1, on the hallmarks of cancer. Moreover, we would like to present the potential of FDFT1 as both a therapeutic target and a biomarker in cancer, and highlight recent trends in the development of FDFT1 inhibitors. Through this, we hope that our review will help to promote research on the development of anticancer drugs that target FDFT1.

## 2. Role of Cholesterol in Normal Cells and Cancer

### 2.1. Role of Cholesterol in Normal Cells

Cholesterol is an essential lipid for maintaining cellular homeostasis [8]. It is mainly synthesized in the liver and transported to the cells of the body through the bloodstream in the form of low-density lipoprotein (LDL) [9]. LDL enters the cell through clathrin-mediated endocytosis, where it is transported to lysosomes, hydrolysed into free cholesterol molecules, and then transported to cell membranes or other cell membrane-bound organelles [9]. Cholesterol homeostasis is tightly regulated by a complex protein network, which functions in transport, synthesis, metabolism, and esterification of cholesterol [8]. Sterol regulatory element binding protein 2 (SREBP2) and liver X receptors (LXR) act as major regulators of cholesterol homeostasis [8]. Cholesterol levels in the endoplasmic reticulum (ER) acts as a sensor for measuring intracellular cholesterol homeostasis. A decrease in ER cholesterol triggers the translocation of SREBP2 from ER to Golgi, and it subsequently goes to the nucleus, activating the transcription of genes involved in cholesterol synthesis, as well as upregulating exogenous entry into the cell. On the other hand, increased cholesterol levels in the cell lower synthesis and promote its excretion through activation of the LXR receptor by oxysterol, an oxidized derivative of cholesterol [10]. Cholesterol is a precursor of steroid hormones and is an essential component of the plasma membrane. Furthermore, it is also enriched in lipids rafts and plays an essential role in intracellular signalling [4,8]. 

Lipid rafts may play an important part in many cellular biological processes including signal transduction pathways, membrane trafficking, cytoskeletal organization, apoptosis, cell adhesion and migration, synaptic transmission as well as pathogen entry [11,12,13]. The most important role of lipid rafts at the cell surface is to regulate receptor-mediated signal transduction because rafts are thought to be physical platforms that provide a distinct efficient microenvironment for interaction between molecules and receptors. Depletion of cellular cholesterol leads to disruption of lipid rafts and release of their protein components into the bulk plasma membrane, ultimately altering signal transduction [14]. Hence, a unique characteristic of lipid rafts is their ability to exclude or include certain proteins, and the same proteins can have different functions inside or outside of membrane rafts.

### 2.2. Role of Cholesterol in Cancer

Changes in cholesterol biosynthesis are regarded as a hallmark of a variety of cancers [15]. Elevated cholesterol levels are involved in a higher incidence of some cancers, such as prostate cancer [16], and breast cancer [17], but colorectal cancer is associated with lower cholesterol contents [18]. Total cholesterol intake is also associated with an increased risk of stomach, colon, rectal, pancreatic, lung, testicular, kidney, bladder, and breast cancer (especially postmenopausal) [19,20]. 

Solid tumours often have a limited vascular supply, and as consequence lipids and oxygen are found at reduced levels in the TME, which could lead to significant upregulation of cholesterol biosynthetic activators such as the master transcription factor SREBP2 and its downstream target genes [21]. As cancer cells undergo rapid proliferation, they depend on elevated cholesterol biosynthesis to supply enough cholesterol for membrane biogenesis and other functional needs such as energy homeostasis, and steroid hormone synthesis.

Cholesterol can contribute to the development of cancer in direct or indirect ways. For example, cholesterol can directly bind to smoothened receptors and activate the hedgehog pathway that causes cancer [22,23]. Cholesterol binds to the adenosine A_2A_ receptor in C6 glioma cells [24]. Intracellular cholesterol is also directly involved in inducing mTORC1 signalling [25]. Lysosomal cholesterol can also activate mTORC1 through the SLC38A9-Niemann-Pick C1 signalling complex [26]. Indirectly, cholesterol can contribute to the development of cancer by facilitating the formation of lipid rafts. Similar to normal cells, many signals for proliferation, migration, and survival of tumour cells are transmitted through cholesterol-enriched lipid rafts [27]. For example, AKT, whose role is critical in the cell survival signalling pathway, may be enhanced when translocated to lipid raft microdomains [28]. The studies demonstrate that reduction of cholesterol level in lipid membrane fractions in cancer cells leads to mechanical disruption of lipid rafts, which therefore activates or inhibits lipid raft-localized proteins. Cholesterol depletion in lipid rafts inhibits the phosphorylation of oncogenes related to rafts such as Akt, c-Met, and Src [5].

Cholesterol is also a precursor to bile acids and steroid hormones, which can initiate or promote colon, breast and prostate cancers [29,30,31]. Many studies have shown that cholesterol mediates its effects on tumour progression through derivatives like cholesteryl esters and oxysterols, which are enriched in the TME. Besides its various direct roles in cancer cells, cholesterol and its metabolites, especially oxysterols, also impact the functions of tumour-infiltrating immune cells in TME, and these include B lymphocytes, T lymphocytes, tumour-associated macrophages, neutrophils, and dendritic cells (DCs).

Oncogenic signals such as PI3K/AKT/mTOR, RTK/RAS and TP53 are also known to regulate cholesterol synthesis in cancer [4]. In prostate cancer, AKT-mediates upregulation of intracellular cholesterol levels and promotes cancer invasion and bone metastases [32,33]. In glioblastoma (GBM), the expression of the LDL receptor was induced by AKT, and pharmacological inhibition of LDL receptor effectively induced cancer cell death [34]. TP53 is the most frequently mutated gene in cancer and is an indicator of poor prognosis [35]. Loss of TP53 function does not regulate the synthesis pathway of cholesterol in breast cancer, which is necessary and sufficient to destroy the structure of breast tissue [36,37]. Reduced activity of ATP binding cassette subfamily 1 (ABCA1) in cancer cells increases mitochondrial cholesterol levels, promoting cancer cell survival [38]. ABCA1 activity is reduced in colorectal cancer cells through mutations that eliminate or reduce its gene expression [38]. Transformation of colon epithelial cells dependent on the expression of mutations in TP53 and RAS decreased the level of ABCA1, and overexpression of ABCA1 in cells modified by TP53 and RAS reduced the growth of xenografted cancer [38]. 

Cholesterol-enriched TME triggered T cell ER stress and increased the proportion of exhausted T cells. CD8^+^ T cells in TME take up and accumulate cholesterol, then upregulate expression of immune surface checkpoints such as PD-1 (Programmed cell death protein 1) and LAG-3 (lymphocyte-activation gene 3) compared with T cells in normal tissues, leading to the dysfunctional state known as functional exhaustion and limited antitumour activity [39]. Decreasing the level of cholesterol in tumours or their microenvironment reduced expression of immune checkpoints on CD8^+^ T cells and restored their antitumour activity [39]. However, cholesterol metabolism is heterogeneous in different tumour-infiltrating T cell subsets. Furthermore, the products of cholesterol metabolism have been shown to damage the function of DCs, which are antigen-presenting cells of the mammalian immune system, in mouse and human tumour models. Cholesterol-enriched DCs are neither able to effectively stimulate allogeneic T cells nor present tumour-involved antigens, resulting in reduced antigen-processing capability. Oxysterols that can activate LXRα signalling in DCs inhibit expression of C-C motif chemokine receptor-7 (CCR7) on the DC surface, thus consequently suppressing the presentation of tumour antigens to T cells [40]. For example, in breast cancer, 27-hydroxycholesterol (27-HC) has been found to guide polymorphonuclear-neutrophils and γδ T cells into the TME but to decrease cytotoxic CD8+ T cells, and in doing so, promote tumour metastasis [41]. Beyond neutrophils, cholesterol also affects the properties of tumour-associated macrophages (TAMs) in TME. Hyaluronic acid oligomers secreted by cancer cells increase cholesterol efflux in TAMs and direct TAMs toward an M2-like phenotype that accelerates cancer development [42].

## 3. Physiological Role of FDFT1

FDFT1 (EC 2.5.1.2, squalene synthase) is a 416-amino acid, 47-kDa enzyme and is found almost exclusively in the ER [43]. The human FDFT1 gene is located on chromosomal region 8p.22-23.1 containing a unique polymorphic inversion [44] and is expressed at particularly high levels in hypothalamus and liver [45]. The FDFT1 human protein sequence shares between 86% and 93% protein identity with other mammals. Furthermore, the presence of FDFT1 not just in vertebrates but also in yeast, bacteria, and plants indicates its critical importance throughout evolution [43,45].

### 3.1. The Roles of FDFT1 in the Cholesterol Biosynthesis Pathway

**Figure 1:**
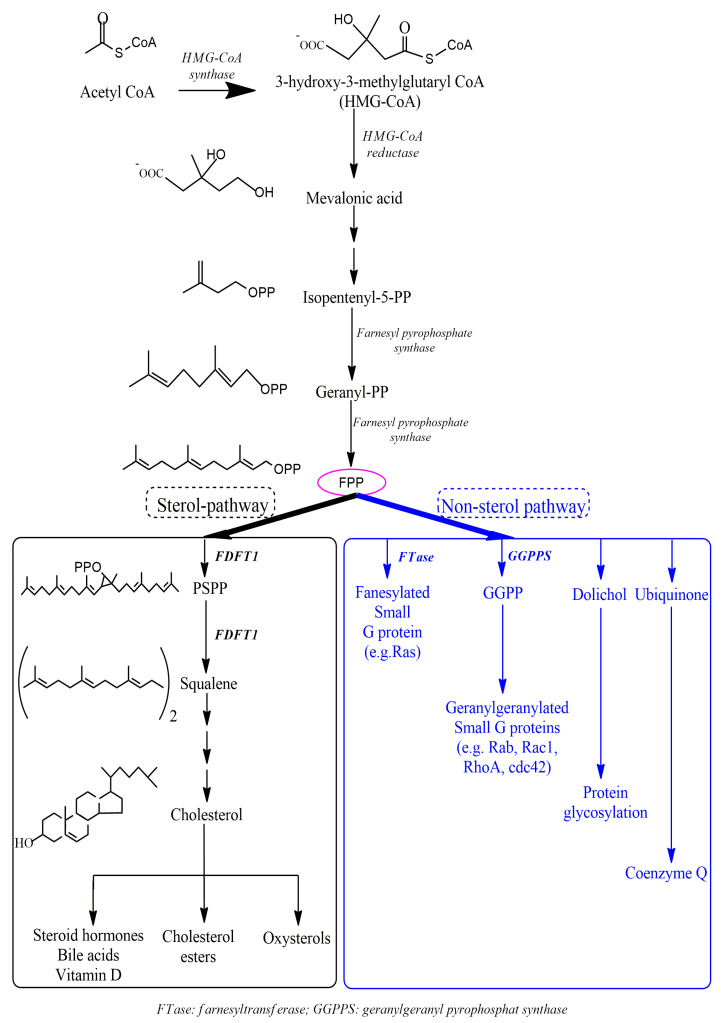
Biosynthesis of cholesterol and its derivatives. Squalene synthase (FDFT1) is the critical enzyme for the synthesis of sterols, and ultimately cholesterol. This enzyme plays an essential role in directing intermediates to either sterol or nonsterol branches of this metabolic pathway [60,61].

FDFT1 is the key enzyme for the synthesis of sterols, and ultimately cholesterol. FDFT1 works in the first stage of liver cholesterol synthesis [46]. FDFT1 is a critical enzyme involved in directing the flow of the metabolite farnesyl pyrophosphate (FPP) to either sterol or non-sterol biosynthetic branches (Figure 1). Mice overexpressing FDFT1 in the liver had elevated cholesterol levels compared to control mice [47]. Because FDFT1 is located downstream in the cholesterol mevalonate pathway than 3-hydroxy3-methyl-glutaryl (HMG)-CoA reductase, the regulation of cholesterol through squalene inhibition does not affect the synthesis of dolichol, ubiquinone (Coenzyme Q10), heme A, and isoprenylated protein [45,48]. FDFT1 is also the first enzyme in this metabolic pathway and catalyses the first reaction directed toward the isoprenoid metabolic pathway and produces squalene via a two-stage reaction. Initially, the dimerization of two molecules of FPP forms pre-squalene diphosphate (PSDP), which is then rearranged and reduced by NADPH to create squalene. The squalene is then converted to cholesterol via a step-multiple process [43]. In the FDFT1 reaction process, a divalent cation, preferably Mg^2+^, is required to bind to and extract a diphosphate group, which is similar to the reaction mechanism of other prenyltransferases [43]. Regulation of FDFT1 activity is thought to play an important role in determining whether FPP is switched toward either sterol or nonsterol branches in response to changing cellular requirements [49,50]. Both ubiquinone and heme A are essential for the mitochondrial electron transport chain [18,51]. Dolichol is a transmembrane carrier for glycol units in the synthesis of glycoproteins and glycolipids. Prenylation is important for post-translational modification and activation of regulatory proteins such as G-proteins, Ras, and p21 [52,53]. Prenylation of G-proteins is required for intracellular signalling between receptors and effector enzymes. Prenylation of Ras protein is an important step in translocation and transformation of Ras, so that it can regulate cell growth [54]. Without the prenylation of these proteins, cell growth is blocked [46]. Besides, oxysterol and farnesyl pyrophosphate, derived from the cholesterol biosynthetic pathway after mevalonate and prior to squalene, may affect the activity of nuclear orphan receptors such as the LXR and farnesoid X activating receptor. These receptors are involved in bile cholesterol metabolism, lipoprotein metabolism, excretion and formation of macrophage foam cells [55]. Therefore, FDFT1 is a key regulatory enzyme in the cholesterol biosynthetic pathway, even though the rate-limiting step of this pathway is catalysed by 3-hydroxy3-methyl-glutaryl-CoA reductase (HMG-CoA reductase) [50].

Besides their roles as structural elements in cholesterol biosynthesis, both products of the FDFT1 reaction, PSDP and squalene have additional functions. PSDP is a bioactive lipid that directly inhibits phospholipase D and leukocyte activities, which leads to down-regulating intracellular signals to decrease the amplitude of the acute inflammatory response and with it the risk of injury to host tissues. This activity suggests that PSDP also displays the properties of a mediator of the inflammatory response in neutrophils [56]. Squalene is converted immediately to squalene epoxide, which then can be directed either into the standard sterol biosynthetic process to form cholesterol, or into the squalene dioxide pathway to form oxysterols such as 24(S), 25-epoxycholesterol [43]. This compound may regulate bile acid synthesis through activation of the LXR [57], suppressing SREBP processing and providing feedback control of cholesterol synthesis [58].

### 3.2. Transcriptional Regulation of FDFT1

**Figure 2:**
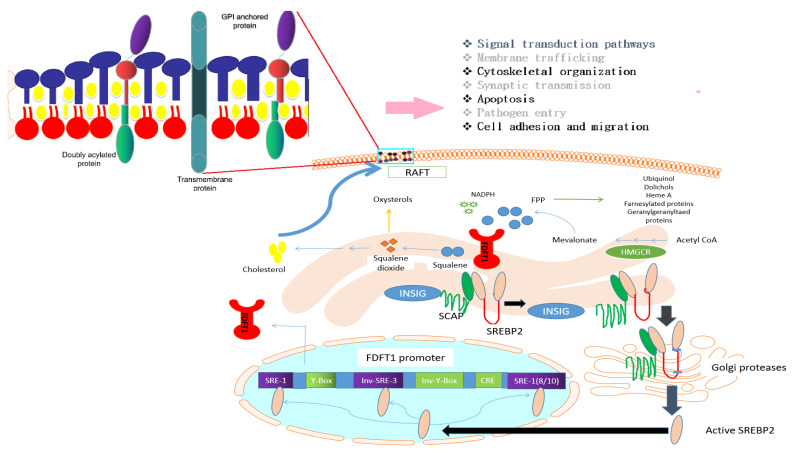
Role of FDFT1 in the biosynthesis of cholesterol, structure and roles of lipid rafts and regulation of FDFT1 by SREBP2. FDFT1 is the first enzyme of the cholesterol biosynthesis branch, as FDFT1 converts farnesyl pyrophosphate (FPP) into squalene which ultimately produces cholesterol and cholesterol derivatives. Therefore, inhibition of FDFT1 only impacts the cholesterol biosynthesis pathway but not an alternative pathway, where FPP is converted into other essential cellular products such as ubiquinol, dolichols, heme A, farnesylated proteins, or geranylgeranylated proteins. Cholesterol is one of the most critical components of the plasma membrane microdomain known as rafts. Beyond cholesterol, the raft is structured by transmembrane proteins, doubly acylated proteins, and glycosylphosphatidylinositol (GPI) anchored proteins. Lipid rafts contribute to a variety of cellular biological processes such as signal transduction pathways, membrane trafficking, cytoskeletal organization, synaptic transmission, apoptosis, pathogen entry, cell adhesion, and migration. One of the key transcriptions factors of FDFT1 is SREBP2. SREBP2 is located in the endoplasmic reticulum (ER) membrane as an inactive form which is bound with Insulin Induced Gene 1 (INSIG) and Sterol Regulatory Element-Binding Protein Cleavage-Activating Protein (SCAP). Upon specific signals such as cholesterol deprivation, PI3K/AKT/mTOR, hypoxia, low pH, androgens, or ER stress, SREBP2 is activated by removal of INSIG from the complex INSIG-SCAP-SREBP2, and then SCAP-SREBP2 is transported to the Golgi, where SREBP2 is cleavage by Golgi proteases to form active SREBP2. The active SREBP2 form translocates into the nucleus and binds to SRE regions in the FDFT1 promoter including sterol regulatory element (SRE)-1, Inv SRE-3, and SRE-1 (8/10), which results in transcription of FDFT1.

The human FDFT1 promoter contains three SREBP-binding sites, sterol regulatory element (SRE)-like sequences, (two SRE-1 motifs and an inverted SRE-3 (Inv SRE-3)), and an auxiliary-binding site for NF-Y (inverted Y-box) (Figure 2), which are essential for the induction of FDFT1 expression by SREBPs. The two SRE-1 sequences and the inverted SRE-3 have been demonstrated to bind SREBP-1 and/or SREBP-2, whereas the NF-Y domain is also related to sterol-mediated regulation of the FDFT1 promoter [1]. SREBPs including SREBP-1a, SREBP-1c, and SREBP-2, are transcription factors for many genes involved in the cholesterol biosynthesis pathway. Evidence suggests that the two SREBP1 isoforms regulate fatty acid and triglyceride synthesis, whereas SREBP2 is a relatively selective activator of cholesterol biosynthesis [59].

SREBPs bind to SRE-like sequences located within 198–127 base pairs (bp) of the transcription start site of the FDFT1 gene. However, SREBPs are only weak transcriptional activators by themselves. The binding of one or more additional transcription factors, such as members of the SP1 (specificity protein 1), NF-Y (nuclear transcription factor Y), CBF (CCAAT-binding factor), and CREB (cAMP response element-binding protein) families, to the promoter in the vicinity of an SRE, is required to stimulate binding of SREBPs to the promoter to stimulate transcription maximally. SREBPs are located in the ER membrane as precursor proteins and are translocated into the nucleus to become the activated form in the presence of activation factors [43], which include, for example, cholesterol deprivation [62], ER stress [63], caspase-3 [64], and the AKT/mTOR pathway in response to decreased growth factors and glucose [30].

In the presence of activation factors, SREBPs are stimulated and distribute from the ER to the Golgi apparatus, leading to subsequent cleavage by site-1 and site-2 proteases to release the active SREBPs, which then are translocated into the nucleus and cause expression of target genes [62,64]. Activation of AKT leads to increased accumulation of nuclear SREBPs. Absence of glucose or inhibition of glycolysis possibly leads to activation of the AMPK (5′ adenosine monophosphate-activated protein kinase) pathway, and consequently, accumulation of nuclear SREBP is blocked in response to AKT activation and prevents expression of SREBP1-induced genes. Furthermore, induction of lipogenic gene expression by SREBP contributes to AKT-dependent cell growth in mammalian cells in vitro and in *Drosophila melanogaster* in vivo [65].

### 3.3. Binding Partners of FDFT1

The action of FDFT1 is regulated or mediated by interacting proteins, and, thus far, 72 have been experimentally proven according to GeneCards and String databases. Among these, we have selected and described the ones that have been best studied, and in particular those that have been reported in articles related to the hallmarks of cancer including proliferation and cell death, metastasis, and cancer metabolism (shown in Table 1). Although interactions between FDFT1 and its partners have been determined by the different methods such as affinity capture-MS and yeast two-hybrid (shown in Table 1), most of these interacting molecules have been not studied for their association with FDFT1 in reports focused on the hallmarks of cancer. Therefore, it warrants further investigation.

## 4. The Role of FDFT1 in Cancer

FDFT1 has been implicated in cancer, both as a potential oncogene and as a potential tumour suppressor gene. While increased gene expression of FDFT1 has been implicated in the development of certain types of cancers [106], the FDFT1gene locus has been observed as the site of deletions in other cancers [107].

Southern blot analysis of oesophageal adenocarcinomas illustrated FDFT1 to be consistently amplified and overexpressed at 8p22-23 in 12.1% of the tumours analysed [106]. Northern blot analysis also showed overexpression of FDFT1 mRNA in all six of the amplified oesophageal adenocarcinomas analysed [106]. FDFT1 overexpression without amplification was not observed [106]. Additionally, loss of heterozygosity [108,109], as well as homozygous deletions [110] in a variety of human cancer cell lines and tumours [107] imply that there are several tumour-suppressor genes on chromosome arm 8p, specifically at the 8p22-23.1 region, where FDFT1 resides [44]. 

The polymorphism of FDFT1 is also associated with cancer. For example, according to the single nucleotide polymorphism database (dbSNP) (http://www.ncbi.nlm.nih.gov/ projects/SNP; build 128), SNPs are found in 244 pieces within 2 kb of the 5′ side and 500 bp of the 3′side of the gene region of FDFT1, but quite a few parts have not been verified yet [45]. The significance of the genetic diversity of the FDFT1 gene in diseases such as cancer and hepatitis C has been partially studied [111]. For example, investigating the SNP rs2645424 polymorphism of this gene in hepatitis C patients, it was found that this polymorphism was associated with progressive fibrosis in patients, not fatty liver [112]. Polymorphisms of FDFT1 are also associated with cancer. This gene has several isotypes. The most common one has eight exons; the promoter of this gene contains the sterol regulatory element (SRE) [45]. In prostate cancer, an association of the rs2645429 polymorphism of the FDFT1 gene with cancer progression and an invasive phenotype was observed. The FDFT1 rs2645429 polymorphism (the rs2645429 allele is reported backwards to the genome) where nucleotide T replaces C is in the promoter of the FDFT1 gene. FDFT1 rs2645429 is located six bp upstream of putative SRE-1, and it has been suggested that the promoter activity of this gene is affected by one base replacement in this polymorphism [113]. Polymorphism has also been reported in lung cancer. The SNP rs2645429 genotype of the FDFT1 gene was examined in patients with NSCLC, showing that the C allele may be a risk factor for this cancer [111]. A significant correlation was found between the presence of the CC genotype in the FDFT1 gene and the risk of NSCLC with overall survival of 3.02 (p = 0 029). Besides, FDFT1 showed copy number aberration in gastric cancer [114]. A mutation of FDFT1 in liver metastasis of colorectal cancer is also related to liver metastasis [115]. 

FDFT1 is highly expressed in liver, lung, prostate, breast, ovary, small intestine, bladder, cervix, thyroid, and oesophageal cancers [116,117,118]. In contrast, FDFT1 expression is downregulated in colorectal, colon, testes, uterus, pancreas, and kidney tumours [118]. FDFT1 has become a potential target in cancer due to its expression as a biomarker for the diagnosis of various cancers [7,116,117]. Conversely, the expression of FDFT1 was downregulated in most of the colorectal cancer (CRC) tumour tissues (19/23) but upregulated in most of the adjacent noncancerous tissues (18/23) and CRC cell lines [7]. 

**Figure 3:**
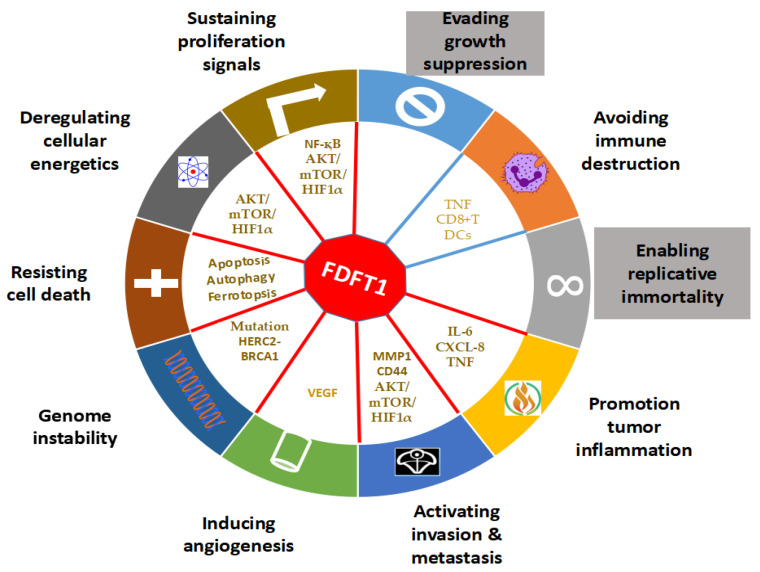
Hallmarks of cancer. Hallmarks of cancer include inappropriately sustained proliferation signals, resistance to cell death, escape from growth suppression, possible replication immortality, invasion and metastasis, inflammation promotion of cancer, avoidance of immune destruction, genetic instability and mutations, and remodelling of cellular metabolism. Seven of the cancer hallmarks originate from tumour cells themselves, while the rest are involved in the tumour microenvironments (TME).

We have summarized how FDFT1 is involved in the development of cancer by dividing it into the functional hallmarks of cancer originating from the cancer cells themselves or the TME (Figure 3). Although attempts were made to understand the role of FDFT1 through its interacting proteins, few studies have been reported on how most interacting molecules participate in FDFT1-involved hallmarks of cancer.

### 4.1. Effects of FDFT1 on Proliferation and Cell Death

Gathering evidence has shown that FDFT1 expression increases in cells undergoing proliferation, suggesting FDFT1 in significant ways, contributes to the maintenance of proliferative signalling in cancer cells via several mechanisms. One of the mechanisms by which FDFT1 promotes tumour growth and proliferation involves enhanced cholesterol biosynthesis. As noted previously, FDFT1 via cholesterol, plays an essential role in the formation and growth of cancer stem cells.

FDFT1 is highly expressed in mammospheres [119], and neuroblastoma sphere-forming cells [120], which are cancer stem cells that exhibit self-renewal and differentiation capabilities as well as cancer therapy-resistant characteristics [121,122,123]. Moreover, high expression of FDFT1 in these cells was demonstrated to relate to advanced stages and lower relapse-free survival [119,120]. Knockdown of FDFT1 significantly reduced mammosphere formation in two TNBC cell lines [119]. Beyond stem cells, down-regulating expression of FDFT1 markedly reduced endogenous cellular cholesterol content, with selective effects noted in raft-associated fractions, resulting in apoptotic sensitivity and cancer cell growth suppression [14,117,124].

FDFT1 regulates cell cycle progression. For example, inhibition of FDFT1 expression significantly attenuated cell proliferation, remarkably increased the sub G1, G1, and G2/M-phase populations, and decreased the S-phase population [14,117,125]. Conversely, supplementation with exogenous cholesterol, increased raft-associated cholesterol and abrogated the suppressive effects on proliferation [124]. These findings suggest that inhibition of FDFT1 and blocking the de novo cholesterol biosynthesis pathway may be an attractive therapeutic strategy to eliminate cancer stem cells and inhibit tumour cell growth.

**Figure 4:**
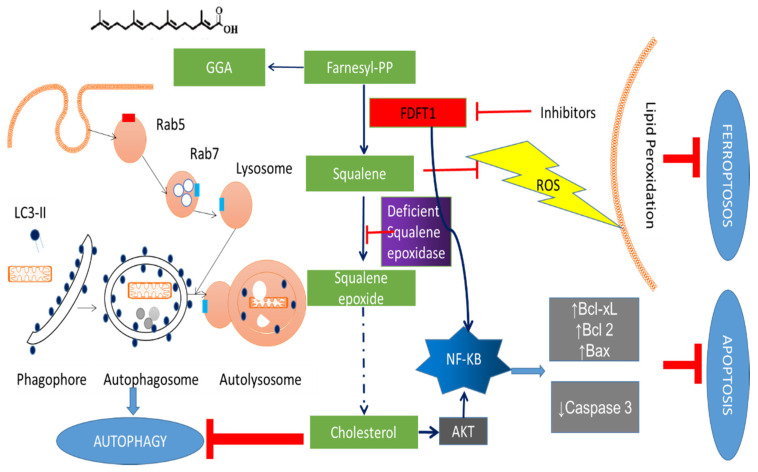
The association between FDFT1 and cell death. FDFT1 is directly or indirectly associated with apoptotic signals. FDFT1 could directly activate NF-KB pathways or activate AKT through cholesterol synthesis. Activated NF-KB increases anti-apoptotic proteins, such as Bcl-xL, Bcl-2, and Bax and decreases pro-apoptotic proteins such as caspase-3, thereby blocking apoptosis signalling. In cancer cells with deficient squalene epoxidase, high expression of FDFT1 increased intracellular squalene levels, which protects the cell membrane from lipid peroxidation by ROS and further prevents the cell from entering the ferroptosis pathway. Furthermore, FDFT1 inhibition upregulates endogenous geranylgeranoic acid (GGA) content, which has been demonstrated to cause incomplete autophagy, and a decrease in cholesterol level also activates autophagy.

FDFT1 is directly or indirectly associated with apoptotic signals (Figure 4). FDFT1 could directly activate NF-κB pathways [18] or activate AKT through cholesterol synthesis [126]. Activated NF-κB increases anti-apoptotic proteins such as Bcl-xL, Bcl-2, and Bax and decreases pro-apoptotic proteins such as caspase-3, thereby blocking apoptosis signalling. In cancer cells with deficient squalene epoxidase, high expression of FDFT1 increased intracellular squalene levels, which protects the cell membrane from lipid peroxidation by reactive oxygen species (ROS) and further prevents the cell from entering the ferroptosis pathway [127]. Furthermore, FDFT1 inhibition upregulates endogenous geranylgeranoic acid (GGA) content resulting in incomplete autophagy and a reduction in cholesterol level also induces autophagy [128].

The role of FDFT1 in proliferation and cell death are clearly mediated through its interacting partners and include epidermal growth factor receptor (EGFR), progesterone receptor membrane component 1 (PGMRC1), oestrogen receptor 2 (ERβ), pannexin 1 (PANX1), RuvB Like AAA ATPase 1 and 2 (RUVBL1 and 2), synoviolin 1 (SYVN1), Unc-93 Homolog B1 (UNC93B1), and WW domain containing oxidoreductase (WWOX) [67,69,73,93,94,99,100,102,103,129,130].

The EGFR is a tyrosine kinase receptor that is activated to form homodimers by binding growth factors like the epidermal growth factor (EGF) or transforming growth factor (TGF-α) and upon activation induces its intrinsic intracellular protein-tyrosine kinase activity [131]. EGFR exists in cholesterol-enriched membrane fractions. Inhibition of cholesterol upregulation helped cancer cells overcome the development of EGFR tolerance and then induced apoptosis [132]. Overexpression of EGFR was shown to be involved in the development of a majority of epithelial malignancies, including breast cancer, head-and-neck cancer, NSCLC, renal cancer, ovarian cancer and colon cancer [131], because of the sustained production of EGFR ligands in the TME or as a result of mutations in EGFR itself [70,72]. Such overexpression enhances downstream signalling pathways, resulting in more aggressive proliferation in tumour cells, and a decrease in median survival [71,133]. Interruption of EGFR signalling can prevent the growth of tumours expressing EGFR and improve patients’ condition, so not surprisingly, EGFR has emerged as a principal target for therapeutic intervention [134]. 

ERβ is a nuclear receptor. ERβ directly negatively regulates genes encoding key components of cholesterol biosynthesis [135]. ERβ upregulates miR-181a-5p leading to inhibition of all branches of the cholesterol biosynthesis pathway by targeting expression of six genes including FDFT1 [136]. ERβ was identified as a binding protein of FDFT1, suggesting that it has some association with the cholesterol metabolic pathway [73]. Moreover, 27HC, a metabolite of cholesterol, promoted the proliferation of ERβ (+) lung cancer cells via PI3K-AKT activation [74]. However, published data indicate that ERβ1 exerts antiproliferative effects, promotes apoptosis and enhances the efficacy of chemotherapeutic agents in ERβ (+) breast cancer cell lines [74].

PGRMC1 is a gene that codes for the protein that makes up the progesterone receptor on the membrane, which is overexpressed, especially in hormone receptor-positive breast cancer [93]. PGRMC1 not only interacts with FDFT1, and SCD1 (stearoyl-CoA desaturase1), but also increases the expression of these lipid metabolizing enzymes, and activates key oncogenic signalling pathways, such as ERα expression and activation, and EGFR signalling, potentially mediating proliferation and progression of breast cancer cells [93]. 

PANX1 is a structural component of the gap junction. *Panx1^−/−^ApoE^−/−^* mice had body weight, serum cholesterol, and showed reductions in triglycerides and free fatty acids [137]. Panx1, therefore, appears to be involved in lipid metabolism. PANX1 plays a crucial role in several cellular processes, such as immune cell death, cell proliferation, invasion, and migration, apoptosis, and autophagy [138]. During cell death, PANX1 channel releases ATP or UTP as a target signal for immune cells.

RUVBL1 and RUVBL2 are ATPases associated with diverse cellular activities (AAAs) and together form RUVBL1/2 complexes [139]. RUVBL1/2 complex participates in chromatin remodelling, as RUVBLs are essential components of ATP-dependent chromatin remodelling complexes INO80 and SWR1 that have impacts on gene transcription activities, and telomerase activity regulation [140,141]. RUVBL1 and 2 strongly link to oncogenesis, where RUVBLs overexpression is correlated with tumour growth and poor prognosis in many cancer types, including liver, breast, colorectal, and NSCLC [95,96]. Furthermore, there is increasing evidence that RUVBLs depletion can hinder growth and progression of cancer cells in both in vitro and in vivo models [142].

SYVN1 is an ER-associated degradation- associated E3 ubiquitin ligase involved in the degradation of proteins from the ER and has also been called HMG-CoA reductase degradation 1 homolog [143]. About 30% of newly synthesized ER-classified proteins fail to fold correctly [144], and SYVN1 is an essential E3 ligase that constitutes part of the quality control system for proteins present in ER, in a process called ER-associated degradation (ERAD). It is not known why SYVN1 interacts with FDFT1. However, as SYVN1 is involved in the decomposition of HMG-CoA reductase, there is the possibility of a role in degrading FDFT1 by recognizing it as a substrate. SYVN1 enhances the ubiquitination and degradation of tumour suppressor p53, which leads to upregulation of cancer cell proliferation and induction of cell death [101]. 

UNC93B1 can interact with the Toll-like receptors TLR3, TLR7, and TLR9, and appears to be involved in the intracellular migration of these receptors within the cell [108]. Therefore, this protein plays an essential role in innate and adaptive immunity by regulating nucleic acid (NA)-sensing Toll-like receptor (TLR) signalling [145]. Interestingly, platelets TLR1, TLR3, TLR6, and TLR7 in women were associated with body mass index, and TLR5, TLR7, and TLR10 were associated with the ratio of total cholesterol to high-density lipoprotein [146]. UNC93B1 promotes tumour growth by regulating the secretion level of granulocyte macrophage colony-stimulating factor in human oral cancer [104]. 

WWOX is an enzyme that contains two WW domains and a short-chain dehydrogenase/reductase domain (SRD). This expression pattern and the presence of the SRD domain suggest a role for this gene in steroid metabolism. WWOX disruption alters high-density lipoprotein (HDL) and lipoprotein metabolism through multiple mechanisms and may explain the low HDL phenotype observed in families expressing WWOX variants [147]. WWOX is a well-known tumour suppressor that affects genetic instability, apoptosis and growth [148,149]. WWOX resides in one of the most common fragile sites known as FRA16D, a region that is altered in many types of cancer [150]. WWOX can act as a tumour suppressor not only owing to its common loss in many human malignancies but also due to its tumour suppressive effect when overexpressed and the susceptibility to tumour formation in WWOX-mutant mice [151,152].

### 4.2. Effects of FDFT1 on Genomic Instability

Maintenance of genomic stability is essential for cellular integrity [153]. DNA replication, endogenous genotoxic stress cell metabolism, such as reactive oxygen species (ROS), and exogenous carcinogenic insults; for example, UV rays, ionizing radiation, or chemicals that damage DNA. Tumour initiation and genomic alterations acquired within the original normal cells lead to the more aggressive selection of subclones [153,154].

The biosynthesis of cholesterol is activated by p53, which suggests that it has some relationship with the function of regulating genomic instability by p53 [3,30]. Accordingly, FDFT1 is one of the genes found to cause spontaneous DNA damage due to knockdown [155,156]. No direct role has been reported for FDFT1 in genomic instability, however, some of FDFT1’s interaction partners, such as a HECT domain E3 ubiquitin ligase (HERC2), nuclear receptor subfamily 2 group C member 2 (NR2C2, Testicular nuclear receptors 4 (TR4)), RUVBL1, and WWOX, are known to play an important role [81,85,94,130]. 

HERC2 is one of the largest genes in the vertebrate genome [157]. HERC2 is involved in the cellular response to DNA damage and takes part in the control of nucleotide excision repair by ubiquitination and proteolysis of XPA (Xeroderma pigmentosum A) that is one of the six core factors of the human nucleotide excision repair system [158,159]. HERC2 facilitates the formation of the RNF8-UBC13 scaffold to recruit BRCA1 to DNA damage sites. Low levels of expression of both HERC2 and BRCA1 decreased the risk of progression and death in advanced NSCLC patients treated with first-line platinum-based chemotherapy [82]. HERC2 ubiquitinates BRCA1 and expresses in breast epithelial cells, and breast carcinomas, indicating a function for HERC2 in breast carcinogenesis [160]. HERC2 interacts with p53, which influences the activation of p53 oligomerization and transcriptional activity through the p53 tetramerization domain [161].

NR2C2 is classified as an orphan receptor. NR2C2 binds to hormone response elements (HREs) located in the promoter of its common downstream target genes, which relates to fertility, neuron development, metabolism and control of lipid uptake in macrophages [162]. NR2C2 induces apoE expression in HepG2 cells [163]. NR2C2 is involved in the expression of CYP3A4, which is involved in the biotransformation of atorvastatin and various effects of statins [164]. Mice lacking NR2C2 exhibited an early ageing phenotype, suggesting that the loss of NR2C2 may be due to genomic instability [165]. 

Depletion of the RuvBL1-RuvBL2 complex in human cells triggers characteristic signs of Fanconi anaemia (FA), including DNA crosslinker sensitivity, chromosomal instability, and defective FA pathway activation [166]. Besides, the expression of RUVBL1 was high in the clinically aggressive micropapillary strain of bladder cancer [167]. PRMT5 (protein arginine methyltransferase 5)-dependent methylation of the TIP60 coactivator RUVBL1 is a crucial regulator of homologous recombination [168]. RUVBLs are also involved in DNA repair and apoptosis via formation of the TIP60 complex to interact with and regulate transcription factors such as Myc, E2F1, and β-catenin [97,169]. 

WWOX deletion in mouse B cells also leads to genome instability [170]. Besides, somatic loss of WWOX is associated with TP53 perturbation in basal-like breast cancer [18]. Therefore, whether FDFT1 can regulate genomic instability through partnerships with these interactions and how it affects cancer may be worthy of further study.

### 4.3. Effects of FDFT1 on Invasion and Metastasis

Tumour metastasis is a significant contributor to the death of cancer patients. FDFT1 is significantly upregulated in high metastatic potential cancer cell lines and higher-grade cancer. Overexpression of FDFT1 as well as its interaction partner, CYP51A1 (cytochrome P-450, family 51, subfamily A), caused an increase in migration/invasion capabilities of cells in vitro and an increase in the number of detectable metastatic lung foci compared with the control animals in vivo, suggesting a positive association between FDFT1, CYP51A1 and invasiveness of lung cancer cells [10]. Upregulation of FDFT1 might enrich tumor necrosis factor receptor 1 (TNFR1) in cholesterol-enriched microdomains within the membrane, leading to enhanced nuclear factor-κB (NF-κB) activation. This activation would result in enhanced matrix metallopeptidase 1 (MMP1)—a critical protease for metastatic cancer and thereby cancer progression. In contrast, invasion, migration, and metastasis of cells are significantly inhibited by loss of function or inhibition of FDFT1, and the number of lung metastases is markedly decreased in mice carrying FDFT1-knockdown tumours [116]. 

The process of membrane protrusion driven by localized polymerization of actin filaments on the submembrane is the first in a multistep process toward carcinoma metastasis [171]. Interestingly, FDFT1 may promote cell migration via this mechanism. Many studies show the contribution of membrane cholesterol in regulating cell shape, adhesion, and motility. The acute depletion of plasma membrane cholesterol increased the spread and motility of cells on fibronectin and decreased their adhesion to fibronectin, following rearrangements to the actin cytoskeleton. Cholesterol depletion by interfering with FDFT1 seems to induce CD44 shedding and delocalization of the focal adhesion complex from rafts, thereby suppressing tumour cell migration and invasion [172]. Therefore, cholesterol content may have a specific effect on the signalling pathways that are significantly involved in regulating cell motility on fibronectin and reorganization of the actin cytoskeleton [173]. A decrease in expression of FDFT1, elevated the expression of zyxin, Wiskott–Aldrich syndrome protein family member 2 (WASP2), as well as LIM and Src homology 3 domain protein 1 (LASP1) [124], which regulates focal adhesion assembly and actin cytoskeleton reorganization [174]. The contribution of zyxin to actin-polymerization may reflect an enhanced interaction between membrane and cytoskeleton [175], resulting in limitations to cell migration upon inhibition of FDFT1.

In contrast, FDFT1 overexpression inhibited cell invasion in CRC. The low expression of FDFT1 was significantly associated with tumour size, histological type, lymph node metastasis, tumour differentiation, invasion, malignant progression, and higher TNM stage [7]. Knockdown of FDFT1 led to a dramatic increase in migration distance and Matrigel invasion [115]. 

Among FDFT1 interacting proteins, those related to metastasis include CD74, Fibronectin 1 (FN1), ERβ1, hexamethylene bis-acetamide (HMBA)-inducible protein 1 (HEXIM1), NR2C2, PANX1, and SYVN1 [66,67,73,75,85,99,100]. CD74, also known as HLA-DR (Human Leukocyte Antigen – DR isotype) antigens-associated invariant chain (Ii), is the invariant polypeptide chain of MHC class II on the cell surface and is involved in the formation and transport of this protein. CD74 was initially indicated to take part in an antigen presentation [176]. The interaction between FDFT1 and CD74-CD44 could also facilitate cell migration, as CD74-CD44 combination increases phosphorylation of the actin severing protein cofilin to promote actin polymerization and F-actin formation, causing more efficient migration and invasion of cancer cells [68]. ER β1 inhibited EMT and invasion, and increased E-cadherin in the TNBC cells [177]. 

FN1 regulates interactions between cells and the extracellular matrix and plays a crucial role in cell adhesion, migration, growth, and differentiation [178]. FN1 is a ligand for various members of the integrin family, and FN1 is also related to the formation and development of numerous tumours. For example, FN1 binds its integrin receptor α5β1 leading to stimulation of the PI3K/AKT pathway [179]. Cholesterol depletion lowered levels of phosphorylated FAK, ERK1/ERK2, and kinase activity of MAPK, which was rescued only on fibronectin by cholesterol supplementation into the cell membrane. FN1 is a gene related to total cholesterol and one of the most essential [180]. FN1 has been demonstrated to promote cell growth and invasion in oesophageal squamous cell carcinoma, oral squamous cell carcinoma, nasopharyngeal carcinoma, and in colorectal, ovarian, renal, gastric and thyroid cancers [76,77,78,79,80,181,182].

HEXIM1 is an inhibitor of RNA polymerase II transcription elongation. HEXIM1 heterozygosity stabilized atherosclerotic plaques, and reduced steatosis in *ApoE* null mice fed an atherosclerotic diet [183]. HEXIM1 selectively regulates the expression of C/EBP and peroxisome proliferator activating receptor-γ (PPAR γ) in skeletal muscle and adipose tissue while adapting to the leptin-mediated metabolic stress signalling pathway in the hypothalamus [184]. Elevated HEXIM1 expression induced differentiation and inhibited proliferation and metastasis of cancer cells [83]. 

NR2C2 alters miR490-3p/vimentin signalling to promote clear cell renal cell carcinoma metastasis [86]. Besides, NR2C2 is as a marker of metastasis, even in non-small cell lung cancer [87]. The NR2C2 also increased prostate cancer invasion by altering TGFβR2/p-Smad3 signalling by decreasing miR-373-3p expression [88] and further, promotes prostate cancer metastasis through upregulation of CCL2/CCR2 signalling [89]. Opposite roles have been reported in liver cancer and other cancers. NR2C2 inhibits HCC cell invasion by down-regulating EphA2 (Ephrin Type-A Receptor 2) expression [185]. 

There are also reports on cancer cell invasion and metastasis of PANX1 and SYVN1. PANX1 promotes invasion and migration are probably related to ERK1/2 kinase activity [186]. Knockdown of SYVN1 inhibited invasion and migration of colon cancer cells via downregulating the expression of MMP-1 and MMP-9 in lung cancer [6,187]. Inhibition of SYVN1 expression resulted in suppression of cancer growth, invasion and migration in hepatocellular carcinoma through an increase in expression of PTEN (Phosphatase and Tensin Homolog) [188]. 

### 4.4. Effects of FDFT1 on Cancer Metabolism

Cancer cells, through a series of mutation-induced metabolic transformations, cause loss of function of tumour suppressor genes, the improved function of oncogenes, increased glucose consumption, decreased mitochondrial respiration, production of reactive oxygen species, and increased resistance to apoptosis [189]. In particular, the accumulation of cholesterol in the mitochondria of cancer cells can contribute to protecting cancer cells from mitochondrial cell death due to changes in mitochondrial membrane dynamics, while partially stimulating aerobic glycolysis to meet the energy demand of proliferating cells. FDFT1 has frequently been found to be upregulated in cancers [118] and to have significant effects on metabolic processes in cancers [6,7]. YM-53601, a specific squalene synthase inhibitor, reduced the mitochondrial cholesterol levels in both H35 and HepG2 cells [190].

**Figure 5:**
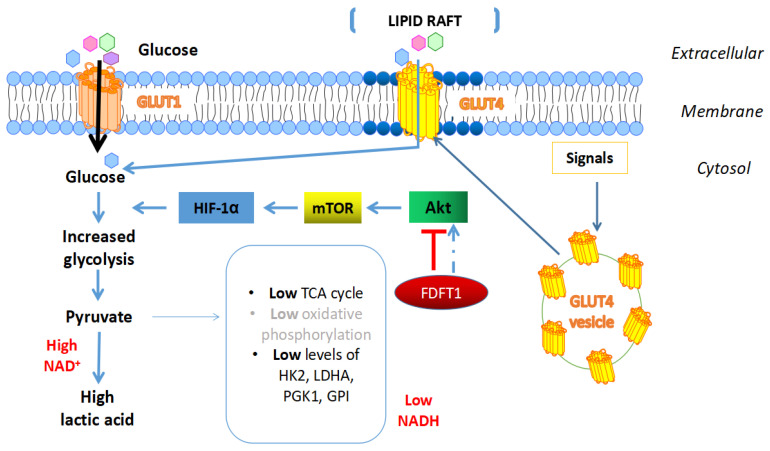
Association FDFT1 with metabolism in cancer. FDFT1 increases cholesterol in membrane microdomains, and thus traffics GLUT4 from the cytosol to lipid rafts in the cell membrane, consequently enhancing glucose uptake from TME into cells. On the other hand, FDFT1 has been determined to inhibit the Akt/mTOR/HIF-1α axis, thereby decreasing expression of glycolytic enzymes such as HK2, LDHA, PGK1, GPI, and ultimately, reducing the lactic acid concentration in the TME. However, this mechanism has only been shown in colorectal cancer [7], and it will be necessary, therefore, to determine whether FDFT1 could inhibit or activate Akt/mTOR/HIF-1α signalling in other cancers.

FDFT1 can inhibit glucose metabolism by suppressing the AKT-mTOR-HIF-1α pathway in CRC [7]. Moreover, overexpression of FDFT1 in CT26 and SW620 cells inhibited transcription and expression of rate-limiting enzymes in glucose metabolism (Glucose transporter 1 (GLUT1), hexokinase 2 (HK2), lactate dehydrogenase A (LDHA), phosphoglycerate kinase 1 (PGK1), and glucose phosphate isomerase (GPI)), leading to reduced glucose uptake and lactate production (Figure 5). These are two primary indicators of the Warburg effect, reducing the extracellular acidification rate (ECAR), which is another indicator of glycolysis but increasing the oxygen consumption rate (OCR), which reflects mitochondrial respiration. Patients with high levels of FDFT1 and a low expression of AKT1, mTOR, HIF-1α, GLUT1, and HK2 exhibited more prolonged survival than those with the opposite expression pattern of low FDFT1, and high expression of genes belong to the AKT-mTOR-HIF-1α pathway and glycolysis [7].

Among the proteins interacting with FDFT1, those related to cancer metabolism include EGFR, FN1, sodium-taurocholate co-transporting polypeptide (SLC10A1), and WWOX [67,69,75,130]. EGF promotes the Warburg effect, inducing epithelial-mesenchymal metastasis and cancer stem-like cell properties in human oral cancer cells [191]. EGFR activates glutamine dehydrogenase 1 transcription to promote glutamine metabolism through the MEK/ERK/ELK1 pathway in GBM [192]. Effective inhibition of EGFR signalling in NSCLC cells leads to a dramatic decrease in the levels of hexokinase II and phosphopyruvate kinase M2 as well as upregulation of the mitochondrial complex subunit [193]. Migration stimulating factor, a genetically truncated N-terminal isoform of fibronectin, promotes tumour growth by reprogramming myofibroblasts to produce lactic acid [194]. 

Sodium-taurocholate co-transporting polypeptide (NTCP, SLC10A1) integrates with membrane glycoproteins to participate in the enterohepatic circulation of bile acids. It was noted that SCL10A1 localizes mainly in lipid rafts and that altering membrane cholesterol concentration resulted in a shift of a substantial proportion of SLC10A1 from rafts, accompanied by changes in the function of the transporter [195]. Inhibition of SLC10A1 in mice increased excretion of biliary cholesterol and phospholipid, without affecting output rates of biliary bile salt, which provides an important elimination route for excess cholesterol [196]. Cholesterol treatment led to increased levels of SLC10A1 and OCT-1 mRNAs [197]. The S267F variant was also associated with decreased risk of the development of advanced liver cirrhosis (LC) and hepatocellular carcinoma (HCC) [198]. SLC10A1 is one of the genes that inhibit HCC cell proliferation by down-regulating glycolysis [98]. 

WWOX is involved in cancer metabolism through glucose metabolism and regulation of HIF-1α levels and functions [199,200]. However, none of these interacting molecules has been studied for their association with FDFT1 in reports focused on cancer metabolism.

### 4.5. Effects of FDFT1 on Angiogenesis and Inflammation

Angiogenesis is a key requirement for tumour growth and metastasis. To date, there has not been much evidence for an impact of FDFT1 expression on angiogenesis. However, in vivo and preclinical evidence indicates an important role of cholesterol in the regulation of angiogenesis for cholesterol transport and distribution to the whole body [201]. 

Inhibition of cholesterol synthesis could block angiogenesis by decreasing vascular endothelial growth factor (VEGF) levels, interfering with VEGF receptor 2 (VEGFR2) dimerization on the endothelial cell membrane and inhibition of endothelial migration, thereby inhibiting signalling and angiogenesis in vitro and in vivo [201,202].

Inhibition of FDFT1 lowered cellular cholesterol and increased the concentration of the isoprenoids FPP and geranylgeranyl diphosphate, which led to potent reductions in IL-6 and CXCL8 secretion from endometrial cells and a reduced inflammatory response [203]. The effects on the inflammatory response by FDFT1 inhibition were as effective as dexamethasone. Moreover, the cholesterol-enriched microdomains are thought to be a signal transduction platform that concentrates cytokine receptors spatially. Therefore, FDFT1 could regulate cytokine responses in a wide variety of cell processes and possibly be a potential target for regulating inflammation [204].

Among the interaction partners of FDFT1, ANXA5, CD74, HEXIM1, PANX1, SLC10A1, SYVN1, UNC93B1, and WWOX are partner molecules involved in angiogenesis and inflammation [66,67,99,100,102,103,130]. ANXA5, also known as thromboplastin inhibitor V, is a calcium- dependent phospholipid-binding protein which is implicated in endocytosis and exocytosis, phagocytosis and autophagy [205]. ANXA5 interacts with glycerophospholipid microcompartments [206]. ANXA5 is elevated in conditions of a high-fat diet [207]. Interestingly, ANXA6, a member of the same ANXA family, is involved in the regulation of EGFR signalling and cholesterol homeostasis [208]. ANXA5 inhibits cyclooxygenase-2 expression by downregulating the protein kinase C-ζ-nuclear factor-κB signalling pathway in prostate cancer cells [41]. 

Inhibiting the expression of CD74 in a bladder cancer cell line (HT-1376 cells), suppressed the expression of VEGF, an angiogenesis inducer [209]. HEXIM1 inhibited tumour angiogenesis via downregulation of HIF-1α expression [84]. PANX1 channel in endothelial cells is involved in vascular inflammation [210]. Genetic deletion of SYVN1, an E3 ligase important for suppressing the ER stress response, increases the expression of the ER stress response gene in Tregs and significantly reduces the ability to suppress Tregs under inflammatory conditions [211]. SYVN1 directly interacts with the deubiquitinating enzyme Usp15. Unlike the classical function of SYVN1 in ER-related degradation, Usp15 does not degrade but loses deubiquitination activity against IκBα deubiquitination resulting in excessive NF-κB activation [212]. UNC93B1 orchestrates TLR7 and TLR9 trafficking to limit lethal systemic inflammation [145]. WWOX expression was inversely correlated with CD31 expression in tumour samples [213]. Reduced WWOX expression promotes angiogenesis in osteosarcoma [214]. 

### 4.6. Effects of FDFT1 on Immune Evasion and Neuronal Contribution

To date, there is no direct evidence showing an association between FDFT1 and immune evasion in tumours; however, it may contribute to suppression of immunity in the TME by mediating cholesterol biosynthesis. Inhibition of FDFT1 in tumour-bearing mice by zaragozic acid decreased cholesterol leading to reduced oxysterol levels within the TME [215]. This partly abrogated LXR activation, decreased neutrophil infiltration, restored DCs functionality and antitumour immune responses were vigorously increased by both the number and the tumouricidal ability of antitumour T cells which released IFNγ and delayed tumour growth. Treatment with combined zaragozic acid and active immunotherapy significantly increased the overall survival of tumour-bearing mice compared to treatment with zaragozic acid alone [215]. 

Among FDFT1s interaction partners, CD74, and UNC93B1 are partner molecules involved in immune evasion. CD74 is significantly high and might prevent the presentation of tumour antigens in various tumour cells [68,216,217]. The interaction of CD74 and MIF regulates the expression of programmed apoptosis ligand 1 in melanoma cells [218]. The physical interaction between UNC93B1 and TLRs is essential for immune TLR signalling, a cascade leading to tumour evasion from immune surveillance [219,220]. Thus, there is a possibility that the interacting proteins of FDFT1 mediate possible immune evasion by FDFT1. Based on these lines of evidence, inhibitors of FDFT1 may be promising adjuvants of the antitumour immune responses and should be combined with immunotherapy to treat cancer patients.

Nerves are an essential part of the TME and contribute to tumour progression [221,222]. About FDFT1, no studies have specifically addressed the role of neuronal interactions in TME. However, there are many neurological implications associated with FDFT1 activity. For example, FDFT1 is essential for normal embryonic development, and FDFT1 homozygous knockout mice resulted in embryonic lethality between E9.5 and E12.5, accompanied by severely impaired neural tube closure [223]. Inhibition of FDFT1 by zaragozic acid (squalestatin) reduced cellular cholesterol content, which prevented accumulation of cellular prion protein in three prion-infected cell lines (ScN2a, SMB, and ScGT1 cells) [224] and protected against A beta-induced synapse damage and neuronal death in vitro [225]. These lines of evidence suggest that reducing cholesterol levels by inhibition of FDFT1 is effective in neuronal protection from dysfunction and death in prion diseases and Alzheimer’s disease. A recent report highlighted the nerve-related role of FDFT1 in embryogenesis and morphogenesis because a marked reduction of FDFT1 exhibited abnormal clinical features including structural brain malformations, cortical visual impairment, and profound global developmental delay in three patients [226]. Moreover, FDFT1 could act downstream of neurotrophic factors IGF-1 and bFGF, which together are one of the three molecular groups involved in tumour-neural crosstalk [227]. What is also noteworthy is that these factors are usually secreted by tumours that have an unfavourable prognosis, by potentiating both the growth of cancer cells and neurites.

Based on the evidence, FDFT1 is likely to be involved in cancer growth and progression through regulatory interactions involving the nervous system. Besides, FDFT1 interactor such as ANXA5, CD74, CD79A, DUSP6, EGFR, FN1, NR2C2, PANX1, UNC93B1, and WWOX are involved in neuronal development. In particular, CD74 might be involved in the peripheral neural invasion of pancreatic cancer cells [228].

## 5. FDFT1 Inhibitors as Anticancer Agents

**Figure 6:**
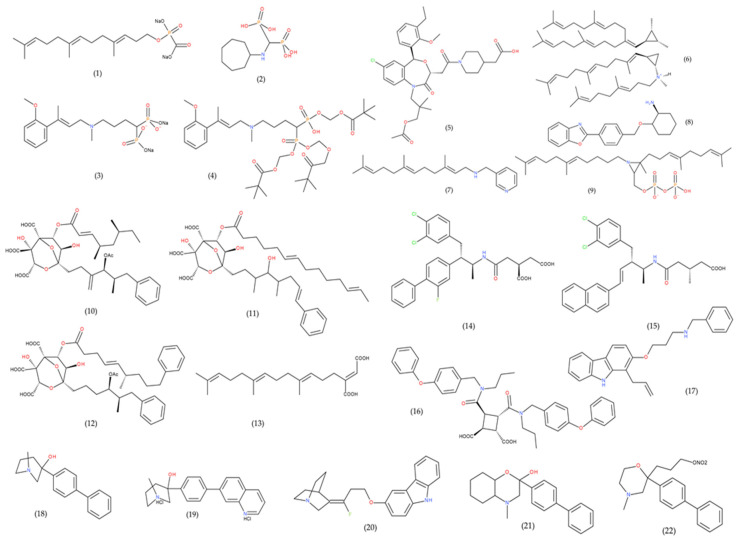
FDFT1 inhibitors. The names of the compounds corresponding to each number are shown in Table 2.

Selective inhibition of FDFT1 only impacts cholesterol synthesis but does not directly interfere with essential products of the non-sterol branch such as dolichols and ubiquinones. Therefore, FDFT1 inhibitors are ideal cholesterol-lowering agents with less toxicity than HMGR inhibitors [117]. To date, many novel inhibitors of FDFT1 have been synthesized and isolated to reduce cholesterol levels. Based on structure, FDFT1 inhibitors fall into several different categories, and these include structural analogues of FPP (the substrate of FDFT1) or pre-squalene pyrophosphate, or transition-state analogues, benzoxazepines, zaragozic acids, dicarboxylic acid and quinuclidine derivatives, as well as substituted morpholine derivatives (summarized in Table 1, Figure 6) [229,230]. Analogues of FPP, the first known inhibitors of FDFT1, were determined to block cholesterol biosynthesis in whole cells. Among FPP analogues, ER-28488 and its pro-drug ER-27856, reduced activity of FDFT1 with IC_50_s of 3.6 nM and 39 μM respectively, potently inhibited cholesterol synthesis both in vitro with rat hepatocytes and in vivo after oral administration to rats. 

Additionally, ER-27856 upregulated LDL receptor activity in HepG2 cells and significantly lowered total cholesterol in plasma [230]. Quinuclidine derivatives suppress the activity of FDFT1 by acting as carbocation mimics of either the first or second step of the conversion of FPP to squalene. Effects on cholesterol reduction by quinuclidine derivatives, such as RPR 107393 and YM-53610 was greater than that of HMG-CoA reductase inhibitors lovastatin or pravastatin [190,231]. 

Moreover, these inhibitors decreased lipogenic biosynthesis in the liver and inhibited secretion of cholesterol and triglycerides from the liver [230,231,232]. Schizostatin, the first dicarboxylic acid derivative isolated from the mushroom Schizophyllum commune, inhibited FDFT1 with IC_50_ 0.84 μM [233]. Besides those, endotoxin, TNF, and interleukin-l decreased hepatic FDFT1 protein and mRNA levels in Syrian hamsters [234]. Furthermore, compounds have been structurally modified to improve the inhibitory potential on FDFT1 activity and absorption after oral dosing. However, in this review, we have only focused on FDFT1 inhibitors that demonstrated their effectiveness in cancer treatment.

Zaragozic acid A is one of three metabolites isolated from fungal sources and has been widely used as a picomolar competitive inhibitor of FDFT1 both in vitro and in vivo. It inhibits by effectively mimicking the binding of presqualene pyrophosphate to FDFT1 [229]. The anti-metastatic effect of zaragozic acid A was tested in CL1-5 lung cancer cell transplanted mice. Mice that received zaragozic acid A at 1 mg/kg displayed the lowest tumour burden, and the lungs of these mice had fewer metastatic nodules than those receiving only saline. Zaragozic acid A reduced the level of photon radiance, lung weights, and the number of surface lung metastases [116]. Inhibiting FDFT1 with zaragozic acid A also attenuated proliferation and induced the cell death of cancer cells [113,115]. Zaragozic acid A was effective but potentially exhibited toxicity due to the over-production of farnesol-derived dicarboxylic acid [235,236]. 

In some studies, the ammonium (“aza”) analogues of the carbocation proved to be a potent inhibitor of FDFT1 by mimicking the electrostatic and topological properties of the putative carbocation intermediates. Moreover, 5-Aza-CR and 5-Aza-CdR are among the many cytosine nucleoside analogues that can inhibit DNA methylation and induce cellular differentiation. Both have been approved by the FDA for treatment of myelodysplastic syndromes and are widely studied for the treatment of haematological cancers, including AML and CML (chronic myeloid leukaemia). Results from quantitative proteomics experiments and Western blot analysis showed that 5-Aza-CdR treatment significantly reduced FDFT1, as the expression ratio (treated/untreated) for FDFT1 was determined to be 0.48 ± 0.10 [124]. 

TAK-475 (a benzoxazepine derivative) is a novel inhibitor of FDFT1 with ED_50_, of 2.9 mg/kg in inhibition of hepatic cholesterol biosynthesis in rats, so TAK-475 has apparent hypolipidemic effects in animals [237]. TAK-475 at high concentration inhibited proliferation of the prostate cancer cell line, PC-3 [113]. Despite these effects, the U.S. Food and Drug Administration (FDA) recommended halting clinical trials of TAK-475 in phases 2 and 3 due to concerns about an increase in levels of the liver enzyme (ALT). There have been recent studies aimed at elucidating the pharmacokinetics, pharmacodynamics, and toxicity of TAK-475 in animals and humans [238,239]. 

Despite this, many novel inhibitors of FDFT1 (summarized in Table 2) have proven effectiveness in reducing cholesterol synthesis and may offer future potential in cancer treatment [237,238,239,240,241,242,243,244,245,246,247]. Among them, YM-53610, ER-27856, and RPR-107393 are novel FDFT1 inhibitors that have demonstrated significant inhibition of lipogenic biosynthesis in the liver as well as secretion of cholesterol and triglycerides [190,230,231,232].

## 6. Conclusions

From the results of multiple studies, FDFT1 could act as an oncogene in some cancers but as a tumour suppressor in colon cancer. No research has yet been done on why this difference occurs. This difference may be due not only to changes in FDFT1 expression but also to differences in how various cholesterol metabolites in cancers result in different responses. It may also be a consequence of the TME exhibiting different responses to FDFT1. These considerations are likely to provide plausible explanations for why FDFT1 acts as an oncogenic gene in some cancers and a tumour suppressor in others.

There are numerous reports on the role of FDFT1 affecting the hallmarks of cancer. However, there has been very little research on the role of molecules that interact with FDFT1. Future research may be productively focused in this area. Finally, although there has been much work on inhibitors of FDFT1, most studies are focused on lowering blood cholesterol, and it seems that more research is still needed on its use as an anticancer agent.

In the future, we anticipate that FDFT1 inhibitors will be used as a new weapon to overcome refractory cancer by overcoming some of these problems.

## Figures and Tables

**Table 1:** Experimental evidence of interaction between FDFT1 and its partners.

Partners of FDFT1	Effects on Cancer Hallmarks	Cancer Types
ANXA5 [66]	Angiogenesis and inflammation	Prostate cancer [41]
CD74 [67]	Metastasis, immune evasion	Breast cancer [68]
EGFR [69]	Proliferation and cell death, cancer metabolism	Breast cancer, head-and-neck cancer, non-small-cell lung cancer (NSCLC), renal cancer, ovarian cancer, colon cancer [70,71,72]
ESR2 (ERβ) [73]	Metastasis	Triple-negative breast cancer (TNBC), lung cancer [74]
FN1 [75]	Metastasis, cancer metabolism	Oesophageal squamous cell carcinoma, oral cell carcinoma, colorectal, ovarian, renal, gastric cancer [76,77,78,79,80]
HERC2 [81]	Genomic instability	NSCLC [82]
HEXIM1 [66]	Metastasis, angiogenesis, and inflammation	Breast cancer, prostate cancer, melanomas, and acute myeloid leukaemia (AML) [83,84]
NR2C2 (TR4) [85]	Metastasis, angiogenesis and inflammation	Clear cell renal cell carcinoma, prostate cancer, hepatocellular carcinoma (HCC) [86,87,88,89]
PANX1 [67]	Proliferation and cell death, metastasis, angiogenesis, and inflammation	HCC, glioma, breast cancer [90,91,92]
PGRMC1 [93]	Proliferation and cell death	Breast cancer [93]
RUVBL1/2 [94]	Proliferation and cell death	Liver, breast, colorectal cancer, NSCLC [95,96,97]
SLC10A1 [67]	Cancer metabolism	HCC [98]
SYVN1 [99,100]	Proliferation and cell death, metastasis	Colon cancer, HCC [101]
UNC93B1 [102,103]	Proliferation and cell death	Oral cancer [104]
WWOX [102]	Proliferation and cell death, cancer metabolism	Ovarian cancer [105]

**Table 2:** Inhibitors of FDFT1 classified by structure.

Structure	Substances	IC50 (FDFT1 Inhibition)	Assay	Reference
**SUBSTRATE (FPP) ANALOGUES**	Isoprenyl Phosphinylformates (1)	8.7–197 μM	Rat liver microsomes	[240]
	YM175 (2)	64 nM	Rat liver microsomes	[231]
	ER-28488 (3)	3.6 nM	Rat liver microsomes	[230]
	ER-27856 (4) (prodrug of ER-28488)	39 μM	Rat liver microsomes	[230]
**BENZOXAZEPINES**	TAK-475 (Lapaquistat) (5)	78 nM	HepG2 cells	[241]
**TRANSITION- STATE ANALOGUES**	Aza analogues (6)	3–10 μM	Yeast microsomes (In the presence of PPi)	[242]
	N-(arylalkyl) farnesylamine derivative (7)	0.05 μM	Rat liver microsomes	[243]
	RPR 101821 (8)	1 nM	Rat liver microsomes	[231]
	Aziridine diphosphate (9)	1.2–1.9 μM	Yeast microsomes (In the presence of PPi)	[244]
**ZARAGOZIC ACIDS** **(SQUALESTATINS)**	Zaragozic acid A (10)	6 μM	HepG2 cells	[229]
	Zaragozic acid B (11)	0.6 μM		
	Zaragozic acid C (12)	4 μM		
**DICARBOXYLIC ACID DERIVATIVES**	Schizostatin (13)	0.84 μM	Unknown	[233]
	J-104,118 (14)	0.52 nM	Hep G2 cells	[245]
	J-104,123 (15)	2.5 nM	Hep G2 cells	[246]
	A-87049 (16)	37 nM	Rat liver microsomes	[247]
**PROPYLAMINE DERIVATIVES**	YM-75440 (17)	63 nM	Hep G2 cells	[248]
**QUINUCLIDINE DERIVATIVES**	ZM-97480 (18)	16 nM	Rat liver microsomes	[249]
	RPR 107393 (19)	0.6–0.9 nM	Rat liver microsomes	[231]
	YM-53601 (20)	79 nM	Hep G2 cells	[190]
**MORPHOLINES**	EP2306 (21)	63 μM	Human liver microsomes	[250]
	EP2302 (22)	1 μM		
